# Taurine Neuroprotection and Neurogenesis Effect in Chronic Ethanol-Induced Rats

**DOI:** 10.3390/nu16121973

**Published:** 2024-06-20

**Authors:** Patricia Rodella, Diogo Boreski, Marcus Alexandre Mendes Luz, Edmo Atique Gabriel, Luiz Fernando Takase, Chung Man Chin

**Affiliations:** 1 Laboratory for Drug Design (LAPDESF), School of Pharmaceutical Sciences, University of São Paulo State (UNESP), Araraquara 14800-903, Brazil; pati.rodella@gmail.com (P.R.); diogo.boreski@unesp.br (D.B.); 2 Advanced Research Center in Medicine (CEPAM), School of Medicine, Union of the Colleges of the Great Lakes (UNILAGO), Sao Jose do Rio Preto 15030-070, Brazil; 30397@unilago.edu.br (M.A.M.L.); edag@uol.com.br (E.A.G.); 3 Morphology and Pathology Department, Federal University of São Paulo of São Carlos (UFSCar), São Carlos 13565-905, Brazil; ltakase@ufscar.br

**Keywords:** taurine, neuroprotection, neurogenesis, hippocampus, ethanol consumption

## Abstract

Taurine (2-aminoethanesulfonic acid) is a non-protein β-amino acid essential for cellular homeostasis, with antioxidant, anti-inflammatory, and cytoprotective properties that are crucial for life maintenance. This study aimed to evaluate the effects of taurine administration on hippocampal neurogenesis, neuronal preservation, or reverse damage in rats exposed to forced ethanol consumption in an animal model. Wistar rats were treated with ethanol (EtOH) for a 28-day period (5% in the 1st week, 10% in the 2nd week, and 20% in the 3rd and 4th weeks). Two taurine treatment protocols (300 mg/kg i.p.) were implemented: one during ethanol consumption to analyze neuroprotection, and another after ethanol consumption to assess the reversal of ethanol-induced damage. Overall, the results demonstrated that taurine treatment was effective in protecting against deficits induced by ethanol consumption in the dentate gyrus. The EtOH+TAU group showed a significant increase in cell proliferation (145.8%) and cell survival (54.0%) compared to the EtOH+Sal group. The results also indicated similar effects regarding the reversal of ethanol-induced damage 28 days after the cessation of ethanol consumption. The EtOH+TAU group exhibited a significant increase (41.3%) in the number of DCX-immunoreactive cells compared to the EtOH+Sal group. However, this amino acid did not induce neurogenesis in the tissues of healthy rats, implying that its activity may be contingent upon post-injury stimuli.

## 1. Introduction

Alcohol consumption is currently an alarming public health issue. Despite its historical use dating back over 7000 years BC in China [1], there are no established safe levels for the consumption of this substance [2]. Alcohol consumption is associated with various conditions, such as hepatic cirrhosis, hepatic fibrosis, cancer, pancreatic diseases, psychiatric disorders [3], diabetes [4], and cardiovascular diseases [5]. The primary damages associated with alcohol consumption are inflammatory, involving the production of chemical mediators such as interleukins (IL-6, IL-8, IL-1B, and TNF-α), TGF-β, and reactive oxygen species (ROS), causing harm to cellular components. Ethanol generally exerts a depressant effect on the central nervous system (CNS), influencing various neurotransmitter pathways and acting directly on various peripheral organs [6]. The intensity of its effects varies according to individual characteristics—such as metabolism, genetic vulnerability, lifestyle, gender, nutritional factors, and duration of consumption—as well as the quantity of substance ingested [7].

Alcohol dependence during detoxification is associated with profound neurobiological and cognitive consequences. Volumetric analyses disclosed noteworthy reductions, reaching up to 10% in bilateral gray matter and up to 20% in the dorsolateral prefrontal cortex. Additionally, albeit less pronounced, decreases were observed in the temporal cortex, insula, and cerebellum. White matter losses, particularly in the corpus callosum, exhibited a scattered distribution. Integration of neuropsychological assessments highlighted a discernible correlation between compromised neuropsychological function and diminished gray matter volume in specific cerebral regions, including the frontal lobe, insula, hippocampus, thalamus, and cerebellum [8]. Concurrently, chronic alcohol consumption induced a reduction in white matter throughout the brain. These findings collectively emphasize the pervasive impact of alcohol dependence on both gray and white matter structures, providing nuanced insights into the intricate interplay between regional neuroanatomical alterations and cognitive performance [9,10].

Animal studies have demonstrated that chronic ethanol consumption induces mitochondrial apoptosis mediated by neuroimmune responses facilitated by cross-talk between neurons and glial cells [11]. The proinflammatory cytokines produced cross the BBB and enhance the effect of ethanol on the CNS, increasing cell death, which may be linked to neurodegenerative diseases, such as Alzheimer and Parkinson [8,12].

The Graham’s Disinhibition Hypothesis (1980) posits that alcohol influences neural regions associated with inhibition and behavioral control, impacting self-regulation, attention, information processing, and decision-making [9]. This theoretical framework suggests that alcohol-induced aggressive behavior may be linked to a narrowed attentional focus, akin to the concept of alcohol myopia, where cognitive processing emphasizes only a limited aspect of the environmental scene, potentially distorting perceptual judgments.

Furthermore, instances of alcohol-related aggression frequently manifest within the context of chronic alcohol consumption and dependence. Empirical studies have estimated that up to 50% of alcohol-dependent men exhibit violent tendencies, with the prevalence varying between 16% and 50%, contingent upon factors such as age and the severity of investigated the violent behaviors [13]. Noteworthy considerations extend to self-harm and suicide [14].

Studies on taurine in the central nervous system were receiving attention as it is a potent antioxidant amino acid that plays a crucial role in neuroprotection by regulating cellular osmolarity, exhibiting antioxidant properties, modulating GABAergic neurotransmission, maintaining calcium homeostasis, and inhibiting excitotoxicity and inflammatory mediators [15,16,17,18,19,20,21,22]. Excessive extracellular glutamate can lead to cellular damage, and taurine has demonstrated neuroprotective effects by reducing intracellular calcium concentration and inhibiting oxidative stress [17]. Due to the broad and diverse activity that taurine has demonstrated, research conducted by Singh suggests that taurine deficiency may serve as an indicator of the aging process [23].

Taurine represents around 0.1% of body weight, with higher concentrations found in excitable tissues such as skeletal muscle, cardiac tissue, the retina, and the central nervous system (CNS). The primary biosynthesis of taurine occurs in the liver via the methionine/cysteine pathway, but it can also be synthesized in other parts of the body, including the CNS. Unlike adults, newborns cannot synthesize sufficient taurine and thus rely on dietary sources, making taurine a semi-essential amino acid. Due to its lack of a carboxyl group, taurine is fully zwitterionic at physiological pH, distinguishing it from most ionizable carboxylic amino acids. This zwitterionic nature imparts high hydro solubility and low lipophilicity, which hinders crossing through biological membranes [24,25].

Despite taurine’s water solubility, its high intracellular concentration is maintained by specific active transporters that concentrate taurine inside cells against its concentration gradient [24]. Taurine absorption is mainly mediated by the TauT transporter (SLC6A6), a member of the sodium- and chloride-dependent SLC6 family of solute carriers located in intestinal microvilli [25]. This family includes at least 16 highly homologous members, such as creatine and neurotransmitter transporters (for GABA, glycine, dopamine, norepinephrine, and serotonin) [26].

Taurine absorption begins with sodium binding to the intramembrane domains of TauT, altering its tertiary structure to facilitate taurine binding and transport. The TauT transporter is Na^+^- and Cl^−^-dependent and has a low transport capacity but high affinity for taurine. After being absorbed in the intestine, taurine is released into the bloodstream through an unknown, non-saturated pathway [25,27]. In the bloodstream, it is distributed to tissues and absorbed by TauT or PAT1 transporters (pH-dependent and Na^+^- and Cl^−^-independent, with low affinity for taurine). It was found that the main uptake occurs via TauT, as evidenced by studies in knockout mice for this transporter, where taurine was reduced by 90% in some tissues [25].

Most taurine biosynthesis is carried out in the liver; however, brain structures such as the hippocampus and cerebellum can also produce it endogenously in small amounts [28,29]. Due to its molecular structure, which possesses a sulfonic acid rather than a carboxylic acid, taurine presents unique physical properties (hydrophilic) compared to other neuroactive amino acids, such as greater difficulty in crossing the blood–brain barrier.

Its concentration in the CNS mainly depends on diet and the complex mechanism of active transport, dependent on TauT, present at the blood–brain barrier [30].

Additionally, the blood–brain barrier expresses the GABA transporter SLC6A13, known as GAT-2, which can also transport taurine across membranes. Both TauT and GAT-2 can efficiently carry hypotaurine as well [31,32]. Deletion of the TauT gene in mice reduces taurine concentrations in plasma and tissues, including the brain. Conversely, genetic deletion of GAT-2 in mice increases brain taurine levels, suggesting that GAT-2 primarily functions as a brain-to-blood efflux system for taurine. TauT is predominantly expressed in astrocytes and, to a lesser extent, in neurons [33].

The synthesis of taurine is highly variable among individuals and is related to nutritional status, protein intake, and cysteine availability [34]. Despite being endogenously produced, a certain amount of taurine is introduced through the diet, primarily in carnivores and, to a lesser extent, in omnivores. The taurine content in foods varies significantly, ranging from less than 1 µmol per 100 g in vegetables, fruits, legumes, and seeds, approximately 20 µmol per 100 mL in cow’s milk, 40 µmol per 100 mL in breast milk, 300–500 µmol per 100 g in beef and pork, to 1000–6000 µmol per 100 g in fish, shellfish, and dark poultry meat [27,35,36].

Taurine is not classified as a neurotransmitter; however, it exhibits various functions that are characteristic of neurotransmitters, such as association with synaptic membrane structures [35] co-localization with its synthesizing enzymes in the presynaptic membrane [28], and reuptake via TauT [30,36]. In the CNS, taurine has numerous functions, most of which are related to neuroprotection, including regulating cellular osmolarity (Morales et al., 2007 [15]), antioxidant action [16], modulating GABAergic neurotransmission [17], maintaining calcium homeostasis inhibiting glutamate-mediated excitotoxicity, inhibiting inflammatory mediators, inhibiting pro-apoptotic proteins, and stimulating anti-apoptotic proteins [18,19,20,21,22].

Various brain areas contain or recapture taurine, such as the pineal gland, hypothalamus, striatum, and cerebellum, promoting neuroprotective action and potentially improving neurodegenerative diseases such as Alzheimer’s disease, Parkinson’s disease, and Huntington’s disease [37].

Studies have shown the importance of taurine in CNS development. Its levels in the immature brain are 3–4 times higher than in the adult brain; this age-dependent decrease can be observed in different species such as mice, rats, monkeys, and humans [38,39,40]. Taurine deficiency resulted in significant deficits in the neural development of cats, rats, and monkeys, and these effects were prevented by taurine supplementation during gestation [41].

In vitro studies using neonatal mouse cell cultures showed the importance of taurine in the early stages of postnatal nervous system development by stimulating cell proliferation and synaptogenesis. Taurine supplementation increased the proliferation of neural progenitor cells in cell cultures and in the dentate gyrus of organotypic hippocampal slices [42].

An in vivo study showed that pre-treatment with taurine reversed this LPS-induced inhibition of neurogenesis in adult rats (WU et al., 2013) [43]. This same study also demonstrated a significant reduction in plasma concentrations of TNF-α and IL-1β in animals that received taurine. Taurine can influence cell survival by modulating the cascade of events leading to apoptosis through the inhibition of pro-apoptotic proteins and stimulation of anti-apoptotic proteins. Some works [21,22,44] showed that taurine increases neural precursor cell proliferation in adult mice. Taurine pre-treatment can reverse LPS-induced inhibition of hippocampal neurogenesis, reducing TNF-α and IL-1β levels. Taurine also influences cell survival by modulating apoptotic pathways. It is hypothesized that taurine could counteract ethanol-induced neurogenesis inhibition and promote neuroprotection through antioxidant, neuromodulatory, and anti-inflammatory effects.

Taurine’s role in neural development is highlighted by its significantly higher levels in the immature brain compared to adults, with deficiency leading to developmental deficits that can be prevented by gestational taurine supplementation [38,39,40,41]. In vitro studies with neonatal mouse cells demonstrated that taurine stimulates cell proliferation and synaptogenesis. Furthermore, exposure to taurine increased neural precursor cells in adult mice, suggesting a role in stimulating cell proliferation through DNA replication.

Given these findings, based on the neuroprotective effects of taurine, including antioxidant action, neuromodulation of GABAergic neurotransmission, and inhibition of excitotoxicity and inflammatory responses, which collectively influence both cell proliferation and survival, we propose that taurine administration holds promise for reversing neurodegeneration induced by chronic ethanol consumption in rats. This study aims to investigate the effects of taurine administration on the hippocampus in a rat model of chronic ethanol-induced CNS injury. Specifically, we examined its impact on hippocampal cell survival, proliferation, and cell death during and after alcohol ingestion. Our findings suggest that taurine administration may have therapeutic potential for reversing neurodegeneration and promoting neurogenesis in a damaged brain.

## 2. Materials and Methods

Wistar rats weighing between 240 and 260 g were employed in this study. The animals were provided by the Central Animal Facility of São Paulo State University (Botucatu-SP). They were transferred to the Pharmacology Laboratory’s Animal Facility within the Department for Drugs and Medicines at the School of Pharmaceutical Sciences in Araraquara, UNESP, at least seven days prior to the commencement of the experiments.

Throughout the study, the animals had ad libitum access to pelleted food and water, and they were subjected to a light/dark cycle of 12 h each. The ethical considerations of this project were endorsed by the Ethics Committee on Animal Use (CEUA) at the School of Pharmaceutical Sciences in Araraquara, UNESP, under protocol CEUA/FCF/CAr 72/15.

### 2.1. Experimental Procedure

The animals were randomly assigned to four groups: H_2_O/Sal (n = 6), EtOH/Sal (n = 6), H_2_O/TAU (n = 6), and EtOH/TAU (n = 6) for each protocol above (total of 48 animals). In the EtOH group, ethanol was provided in bottles with different concentrations over a 28-day period (5% in the 1st week, 10% in the 2nd week, and 20% in the 3rd and 4th weeks) [45]. Animals in the TAU group received daily taurine injections (i.p., 300 mg/kg, diluted in sterile 0.9% saline solution, at a volume of 1 mL/kg). Control group animals (Sal) received injections of the vehicle only (sterile 0.9% saline solution, at a volume of 1 mL/kg). Animals in the control group (H_2_O) were maintained with ad libitum access to water.

**Figure 1:**
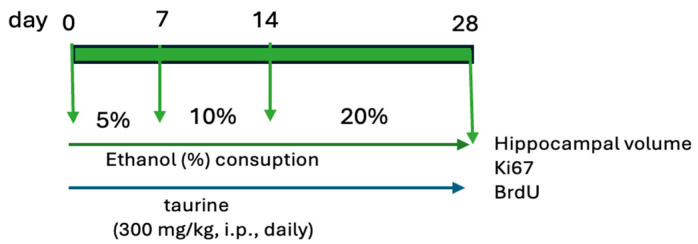
Experimental protocol of taurine neuroprotection effect on chronic consumption ethanol.

**Figure 2:**
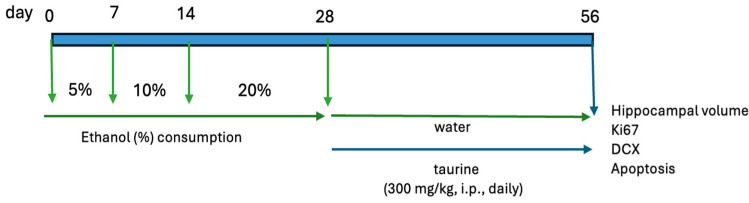
Experimental protocol for the taurine effect on neuronal damage induced by chronic ethanol consumption.

The study was bifurcated into two experiments. The first experiment examined the protective effects of taurine on hippocampal neurogenesis when subjected to the impact of ethanol consumption (Figure 1). The second experiment investigated whether taurine administration could reverse the deleterious effects of ethanol consumption on hippocampal neurogenesis (Figure 2).

#### 2.1.1. Brain Slices

Animals were anesthetized with an i.p. injection of ketamine (150 mg/kg) and perfused transcardially with 0.9% saline, followed by 4% paraformaldehyde in 0.1 M PBS, pH 7.4, at 4 °C. Brains were removed, post-fixed in 4% paraformaldehyde in PBS at 4 °C for 24 h, cryoprotected in 30% sucrose solution in PBS, and cryosectioned into 40 μm coronal sections. The sections were stored in an antifreeze solution at −20 °C.

#### 2.1.2. Hippocampal Cell Volume and Death Analysis

A series of histological sections were stained with cresyl violet (CV) for the analysis of volume and the number of pyknotic cells (cells in the process of neural loss), according to Herrera et al. [46]. The quantitative analysis of pyknotic cells serves as an indicator of apoptosis and cell death. During apoptosis, the cell undergoes morphological changes, including cell shrinkage, chromosomal DNA fragmentation, and chromatin condensation. When these cells are in a shrunken state and associated with hyperchromatosis, they are classified as pyknotic cells. For the quantitative analysis of pyknotic cells, only those with shrunken cell bodies and intensely stained nuclear condensation were considered. Initially, the sections were sequentially mounted on special slides (Superfrostplus Gold, Fisher Scientific, Hampton, NH, USA) and air-dried in an oven at 37 °C for 8 h. The slides were then stained with cresyl violet and coverslipped using DPX (BDH, Gallard-Schlesinger Industries Inc., CarlePlace, NY, USA) as the mounting medium.

**Figure 3:**
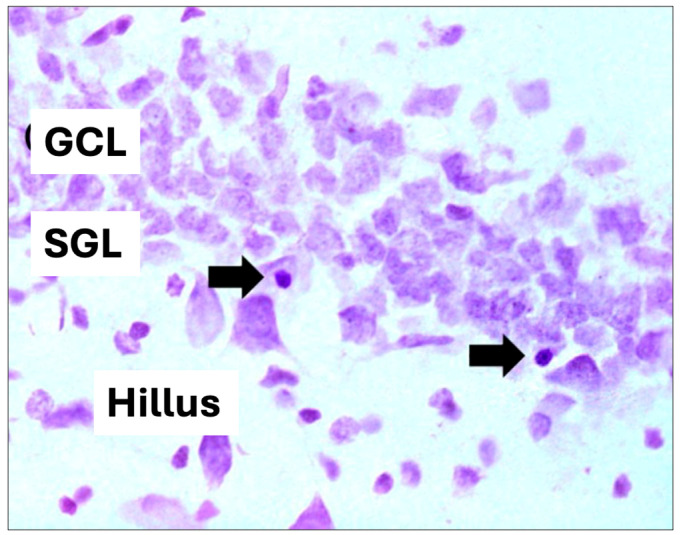
Hippocampal cell volume and pyknotic cell. (GCL): granular cell layer, (SGL): subgranular zone. Arrows indicate pyknotic cells (cell death) (400× magnification).

For hippocampal volume analysis, images were captured using a digital camera attached to the microscope and analyzed using the Axio Vision program (Zeiss, São Paulo, Brazil). The analyzed areas were summed, multiplied by the thickness of the section (40 μm), and multiplied by the distance between sections to calculate the total volume estimate of the structure, expressed in mm^3^, and for the quantitative analysis of pyknotic cells, only those showing a contracted body and intensely stained nuclear condensation were considered), according to [46] (Figure 3). The quantification results represent the average of the sum of the results found in each of the multiple sections of an animal. For statistical analysis, ANOVA and the Bonferroni multiple comparison test were used. A *p*-value < 0.05 was considered significant.

#### 2.1.3. Cell Proliferation, Survival, and Neurogenesis (BrdU, Ki-67, and DCX)

For immunohistochemical processing of Ki-67 (cell proliferation), BrdU (cell survival), and DCX (neurogenesis and cell maturation), histological sections were sequentially mounted on special slides (Superfrostplus Gold, Fisher Scientific, USA) and air-dried in an oven at 37 °C for 8 h. To unmask the antigen, slides were boiled in citric acid (0.01 M, pH 6.0) for 6 min and cooled to room temperature for 20 min. Subsequently, the sections were quickly immersed in distilled water and then washed in PBS. At this stage, slides processed for Ki-67 and DCX were incubated with primary antibodies. Slides were incubated with primary antibody against Ki-67 (mouse-derived, 1:200, Novocastra Laboratories Ltd., Newcastle, UK) in PBS containing 0.5% Tween-20 (Sigma-Aldrich Chemical Co., St. Louis, MO, USA) for 48 h at 4 °C or with primary antibody against DCX (rabbit-derived, 1:500, ABCam, Cambridge, MA, USA) in PBS containing 0.3% Triton X-100 (Sigma-Aldrich Chemical Co., St. Louis, MO, USA) for 48 h at 4 °C.

Slides processed for BrdU underwent additional steps. The tissue was digested in a trypsin solution (0.1% in 0.1 M Tris buffer, pH 7.5, containing 0.1% CaCl_2_) for 8 min. After washing in PBS, the sections were denatured in an acidic solution (2.4N HCl in PBS) for 30 min. After washing, the sections were incubated with a primary antibody against BrdU (mouse-derived, 1:200, Novocastra Laboratories Ltd., Newcastle, UK) in PBS containing 0.5% Tween-20 (Sigma-Aldrich Chemical Co., St. Louis, MO, USA) for 48 h at 4 °C. Following this, sections processed for Ki-67 and BrdU were again washed in PBS and incubated with a biotinylated secondary antibody (horse against mouse, 1:200, Vector Laboratories, Burlingame, CA, USA) in PBS for 120 min at room temperature.

Sections processed for DCX were incubated with biotinylated secondary antibodies (goat against rabbit, 1:200, Vector Laboratories, Burlingame, CA, USA) for the same duration. After washing in PBS, all sections were incubated with the avidin–biotin complex (Vectastain Elite ABC kit, Vector Laboratories, Burlingame, CA, USA) for 90 min at room temperature. The sections were washed with PBS and subjected to the reaction using 3,3-diaminobenzidine (DAB, Sigma-Aldrich, St. Louis, MO, USA) as the chromogen. The reaction was stopped with further PBS washes. Slides were counterstained with cresyl violet and coverslipped using DPX (BDH, Gallard-Schlesinger Industries Inc., Carle Place, NY, USA) as the mounting medium.

### 2.2. Statistical Analysis

The quantitative analysis of neurons immunoreactive to Ki-67, BrdU, and DCX was carried out in a blinded fashion using light microscopy, focusing solely on cells with clearly defined boundaries and conspicuous labeling. The analysis was conducted bilaterally across all sections, spanning the entire dentate gyrus of the hippocampus. The quantification results represent the mean of the total neurons found in each of the multiple sections of an individual animal. Statistical analysis employed ANOVA and the Bonferroni multiple comparison test, with significance set at *p* < 0.05.

## 3. Results

**Figure 4:**
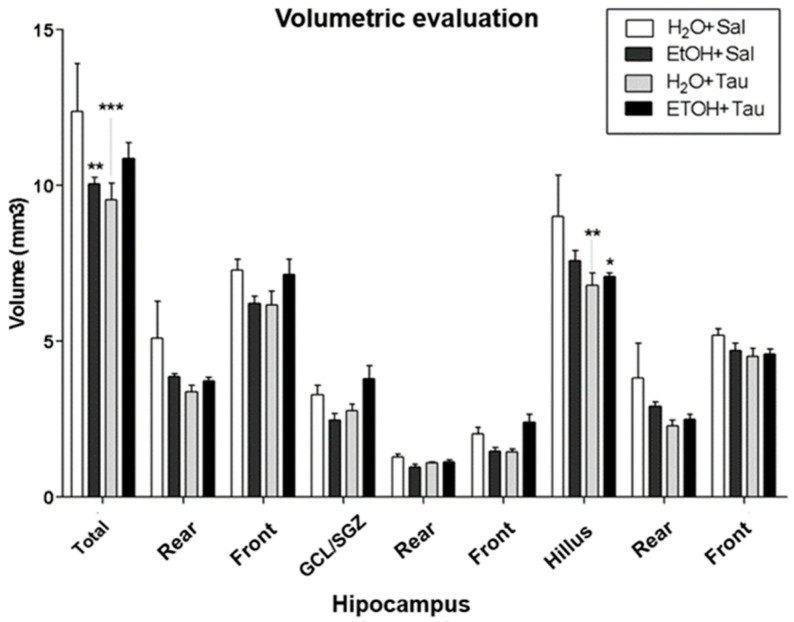
Hippocampal volume profile after chronic ethanol ingestion and taurine (300 mg/kg, i.p) (n = 6). * *p* < 0.05 vs. H_2_O/saline, ** *p* < 0.01 vs. H_2_O/saline, *** *p* < 0.001 vs. H_2_O/saline.

The results showed a decrease in hippocampal volume among rats exposed to chronic alcohol consumption. Volumetric assessment revealed a significant reduction in hippocampal volume across all experimental cohorts compared to the H_2_O/saline (control) group (Figure 4). Specifically, the EtOH/Sal group displayed an 18.8% decrease in dentate gyrus volume. The H_2_O/TAU group also exhibited reductions of 23.0% in the dentate gyrus and 24.7% in the hilus, while the combination of the EtOH/TAU group manifested a volume decrease of 6.9%, limited to the hilus.

### 3.1. Cellular Proliferation—Ki-67

**Figure 5:**
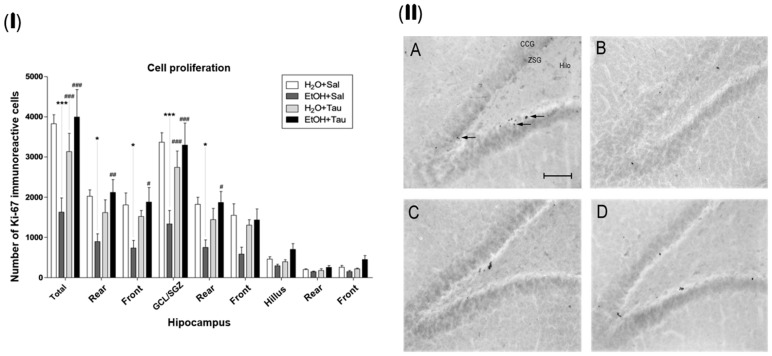
Effect of taurine on hippocampal neuroprotection: (**I**) number of Ki-67 profiles in the dentate gyrus of rats submitted to chronic ethanol consumption (n = 6). (**B**). * *p* < 0.05 vs. H_2_O/saline, *** *p* < 0.001 vs. H_2_O/saline, # *p* < 0.05 vs. EtOH/saline, ## *p* < 0.01 vs. EtOH/saline, ### *p* < 0.001 vs. EtOH/saline; (**II**) pattern of labeling of Ki-67 in the dentate gyrus of rats submitted to chronic ethanol consumption: (**A**) H_2_O/saline group, (**B**) EtOH/saline group, (**C**) H_2_O/TAU group, and (**D**) EtOH/TAU group. (CCG): granular cell layer; (ZSG): subgranular zone. Arrows indicate the Ki-67 immunoreactive cells. Calibration bar: 50 µm.

The gene encoding the Ki-67 protein (MKI67) acts as an intracellular signaling promoter of division and is overexpressed in conditions such as cancer. However, Ki-67 expression is intrinsic to life, playing a crucial role in cellular renewal and the creation of new neural networks [47]. Accordingly, a lower number of Ki-67 immunopositive cells were identified among the experimental groups, suggesting that alcohol significantly reduces the expression of proliferative stimuli in the dentate gyrus cells, with a total decrease of 57.5%. There was a reduction of 55.9% in the rear portion, 59.4% in the front, and 60.5% in the granular cell layer. The results (EtOH+TAU) demonstrated a substantial increase in the number of Ki-67 immunoreactive cells in the entire dentate gyrus (145.8%), in its rear and front portions (137.4% and 155.9%, respectively), and in the granular cell layer (147.5%) and its rear portion (149.5%), Figure 5(I). Figure 4(II) illustrates the pattern of Ki-67 immunoreactive cell labeling in the different experimental groups and shows the image of the slices showing the pattern of labeling of Ki-67 immunoreactive cells in the dentate gyrus of the hippocampus in the different experimental groups. Thus, taurine significantly promoted neuroprotection against the effects of ethanol on cellular proliferation in the dentate gyrus of the hippocampus, surpassing control levels.

### 3.2. Cell Survival—BrdU

Bromodeoxyuridine (BrdU) incorporation assays have been extensively employed for detecting DNA synthesis both in vivo and in vitro. The fundamental concept underlying this technique is that BrdU, when integrated as a thymidine analog into nuclear DNA, serves as a distinctive label that can be traced using antibody probes [48], enabling the analysis of cellular survival in the dentate gyrus.

**Figure 6:**
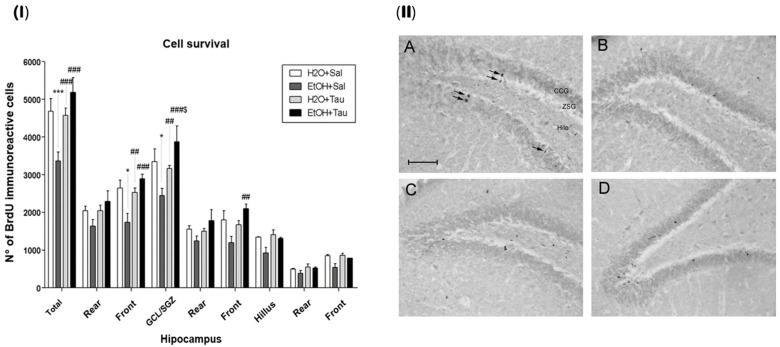
Effect of taurine on hippocampal neuroprotection. (**I**) BrdU number profile of immunoreactive cells in the dentate gyrus of rats subjected to chronic ethanol consumption (n = 6). * *p* < 0.05 vs. H_2_O/saline, *** *p* < 0.001 vs. H_2_O/saline, ## *p* < 0.01 vs. EtOH/saline, ### *p* < 0.001 vs. EtOH/saline $ *p* < 0.05 vs. EtOH/TAU. (**II**) Pattern of labeling with BrdU in the dentate gyrus of rats subjected to chronic ethanol consumption. (**A**)—H_2_O/saline group; (**B**)—EtOH/saline group; (**C**)—H_2_O/TAU group; and (**D**)—EtOH/TAU group. CCG—granular cell layer; ZSG—subgranular zone. Arrows indicate BrdU immunoreactive cells. Calibration bar: 50 µm.

The results demonstrated that the administration of taurine significantly promoted neuroprotection against the effects of ethanol, as shown by the image of the BrdU labeling pattern of immunoreactive cells in the different experimental groups (Figure 6).

The EtOH/saline group exhibited the lowest number of BrdU-immunopositive cells among the experimental groups, indicating a reduction in cell survival in the dentate gyrus of 28.2%, in the frontal portion of 34.3%, and in the granular cell layer (GCL) of 27% when compared to the control (H_2_O/saline). In contrast, taurine led to an increase in cell survival in the entire dentate gyrus (26.4%), its frontal portion (31.3%), and the GCL (22.9%) when associated with ethanol, in comparison to the control. The results suggested that taurine effectively mitigated the deleterious effects of ethanol. There was an observed increase in the number of BrdU-immunopositive cells in the EtOH/TAU group compared to EtOH/saline: dentate gyrus (54.0%), frontal portion (66.6%), GCL (58.9%), and frontal portion (75.1%), all exceeding values observed in the control group (H_2_O/saline), although not statistically significant.

### 3.3. Cell Death

**Figure 7:**
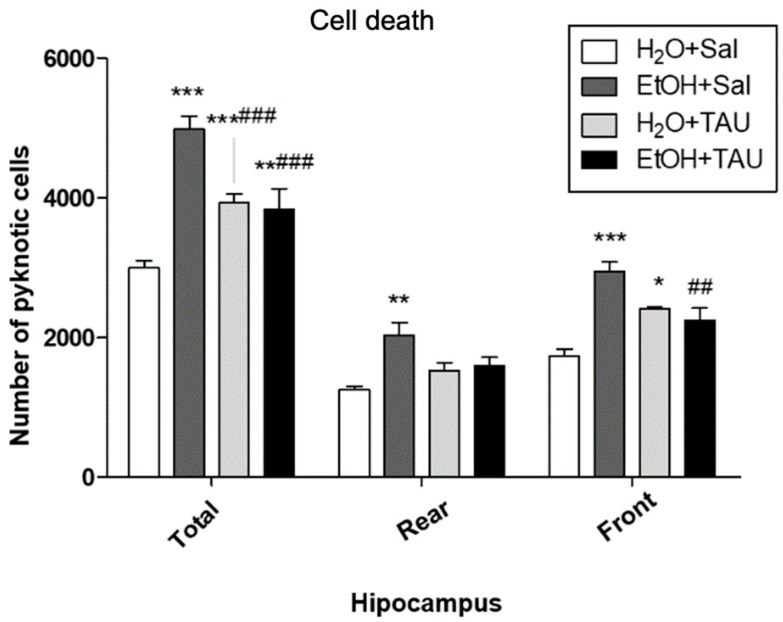
Effects of taurine administration on cell death in the hippocampus of rats subjected to chronic ethanol consumption (n = 6). * *p* < 0.05 vs. H_2_O+Sal, ** *p* < 0.01 vs. H_2_O+Sal, *** *p* < 0.001 vs. H_2_O+Sal, ## *p* < 0.01 vs. EtOH+Sal, ### *p* < 0.001 vs. EtOH+Sal.

In general, all experimental groups exhibited a significant increase in cell death in the dentate gyrus or its anterior and posterior divisions compared to the untreated control group. Quantitative analysis revealed a significant rise in the number of pyknotic cells in the dentate gyrus (66.3%) and in its anterior and posterior portions (62.2% and 69.3%, respectively) in the EtOH/Sal group. Taurine conferred protection against ethanol-induced effects, resulting in a 38.2% reduction in picnotic cells in the dentate gyrus, a reduction comparable to that observed with taurine treatment alone (H_2_O and TAU) (Figure 7).

Chronic ethanol administration also led to a significant increase in cell death in the granular cell layer and a decrease in the hippocampal volume compared to the control group. These findings are consistent with the existing literature. Chronic and excessive ethanol consumption may impair neuronal signal transduction pathways [49].

In vitro studies using rat cortical neuron cultures revealed that ethanol induced a reduction in CREB (cAMP response element binding protein) activity and decreased the expression of brain-derived neurotrophic factor (BDNF), which is essential for neurotransmitter synthesis and other molecules necessary for neuronal survival [50]. These data suggest that neuronal death following ethanol exposure is related to intracellular signaling alteration, the suppression of CREB activity, and decreased BDNF levels [51].

There are few studies on the neuroprotective effects of taurine against ethanol, and to date, these studies have only analyzed the cerebellum of neonatal mice [52,53], which demonstrated the neuroprotective action of taurine in the cerebellar cells of neonatal mice subjected to acute ethanol intoxication. These studies showed a significant reduction in the number of cells immunopositive for apoptosis markers Caspase-3 and Tunel in various cerebellar regions after acute taurine administration in animals receiving subcutaneous ethanol injection. However, they also showed that taurine was unable to neuroprotect Purkinje cells in 4-day-old animals. Thus, the authors suggested that the neuroprotective effects of taurine are not linear, presenting differences depending on the neuronal type studied and the taurine concentration in the plasma. Analysis of the number of picnotic cells through cresyl violet staining can be used as an indicator of apoptosis [54].

### 3.4. Effect of Taurine in the Reversion of Damages Caused by Chronic Ethanol Consumption

**Figure 8:**
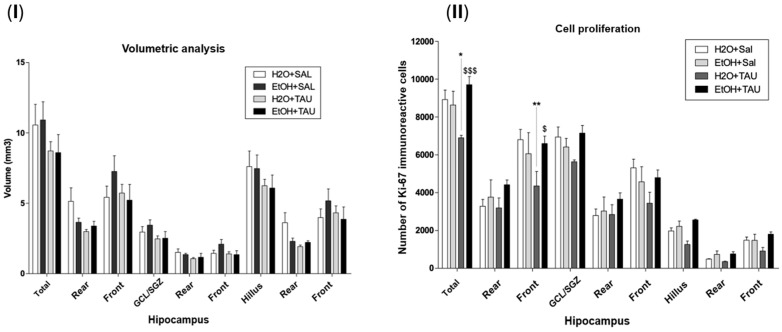
Effect of taurine administration in the hippocampus volume on the reversion of damage in rats induced by chronic ethanol consumption experiment (n = 6). (**I**). Hippocampal volume; (**II**) cell proliferation—Ki67. * *p* < 0.05 vs. H_2_O/saline, ** *p* < 0.01 vs. H_2_O/saline, $ *p* < 0.05 vs. EtOH/TAU, $$$ *p* < 0.001 vs. EtOH/TAU.

Figure 8 shows the results of hippocampal volume and cellular proliferation, Ki67, in the reversion of damage in rats induced by chronic ethanol consumption experiments. In the group of ethanol only, the hippocampal volume was returned to the control value, and no differences were observed for the hippocampal volume with taurine treatment after 28 days of chronic ethanol consumption. The results observed in this experiment demonstrated that after 28 days following the cessation of ethanol consumption, cellular proliferation returned to levels comparable to those of the control group animals who received only water.

### 3.5. Neurogenesis

The assessment of neurogenesis in the dentate gyrus revealed that the administration of ethanol and/or taurine induced alterations in hippocampal neurogenesis. The exposure to ethanol induced a significant reduction in neurogenesis. The ETOH/SAL group exhibited the lowest number of doublecortin (DCX)-immunoreactive cells among the experimental groups; compared to water, it showed a significant decrease in neurogenesis in the dentate gyrus (39.5%) and its frontal portion (51.1%) (Table 1).

Taurine also demonstrated neuroprotective effects against ethanol-induced neurogenesis impairment. The EtOH/TAU group showed a substantial increase in the number of DCX-immunoreactive cells in the dentate gyrus (41.3%) and frontal portion (59%) compared to the control (EtOH/saline). Taurine also significantly reduced neurogenesis in the entire dentate gyrus (19.7%) when compared to the control.

## 4. Discussion

The metabolism of ethanol is linked to the production of reactive oxygen species (ROS). The conversion of ethanol to acetaldehyde generates free radicals, unstable molecular species capable of causing damage to macromolecules essential for cellular homeostasis, such as lipid peroxidation, crosslinks, DNA adducts, and DNA strand breaks. Consequences particularly detrimental to the proper functioning of the brain include mitochondrial dysfunction, altered neuronal signaling, and inhibition of neurogenesis [55].

The decrease in hippocampal volume in animals in the H_2_O/TAU group was unexpected, as it was anticipated that the volume would be at least similar to that of the H_2_O/Sal group. However, this decrease in hippocampal volume did not alter cell proliferation or cell survival in this group.

It is important to highlight that, 28 days after the cessation of ethanol exposure, the hippocampal volumes of the four groups did not show statistically significant differences. These data indicate that this volumetric decrease was transient, suggesting hippocampal recovery.

Due to its important role as an osmolyte, taurine contributes to the control of cell volume [56]. This result suggests that taurine may have reduced cell volume and, consequently, the volume of the hippocampus. Twenty-eight days after the cessation of ethanol intake, the cells and hippocampus returned to normal conditions.

As no behavioral or memory/learning tests were conducted, it is not possible to determine whether this volumetric decrease caused any deficits in the animals. Morphometric studies in aged animals have shown an age-dependent volumetric decrease, yet with the maintenance of cell numbers and neural circuits, suggesting a reorganization of the hippocampus [57,58].

Gebara et al. [59] showed that an increase in cell proliferation or survival does not necessarily result in an increase in hippocampal volume. This finding suggests that a reduction in volume does not necessarily indicate a decrease in cell proliferation or survival.

Studies have demonstrated that, in response to ethanol exposure, the rodent hippocampus undergoes two distinct periods of significant cell proliferation: the first, after two days of abstinence [60], and the second, after seven days [61]. According to these studies, during this second period, there is a fourfold increase in cell proliferation, with survival and neuronal differentiation rates similar to those observed in the control group [61]. These findings suggest that this complex hippocampal self-repair mechanism is responsible for neuronal repopulation in this structure.

The significant reduction in the number of Ki-67 immunoreactive cells observed in our experiments in ethanol-treated animals is supported by literature studies describing a substantial inhibitory effect of ethanol on cellular proliferation in the hippocampus [62,63,64,65]. Contrary to these findings, there are reports of not statistically significant differences after 10 days [66] or 6 weeks [46] of chronic ethanol intake, suggesting that the animals’ organisms may have developed tolerance to its inhibitory effects, although its mechanisms remain unclear at present.

Oxidative stress is an imbalance in the redox system, characterized by excessive free radicals and a weakened antioxidant system. Prolonged alcohol consumption exacerbates oxidative stress, affecting cellular components like proteins, lipids, and DNA [67]. The brain, due to its high oxygen demand and low endogenous antioxidants, is highly vulnerable to oxidative stress. Mitochondria, the primary energy producers, generate over 90% of cellular ATP [68]. The brain’s high phospholipid content, rich in polyunsaturated fatty acids (PUFAs), makes it prone to lipid peroxidation (LPO) [69]. Alcohol-induced oxidative stress leads to LPO, protein and DNA damage, mitochondrial dysfunction, elevated cytokine production, and ultimately neuronal cell death [70].

The neuroprotective effects of taurine and its derivatives have been extensively documented, with particular emphasis on the discovery and enhanced understanding of the Tau-T taurine receptor, which plays a pivotal role in elucidating the potential mechanisms of action of this compound. Taurine has been linked to the mitigation of neuroinflammation in rat models of Alzheimer’s disease, where a notable increase in disease markers was observed. It has been identified as a partial agonist of glycine receptors, leading to the reduction of glutamatergic currents and a decrease in pro-inflammatory cytokines such as interleukins [71]. In the realm of neurodegenerative disorders, taurine supplementation has shown efficacy in decreasing the secretion of inflammatory markers including TNFα, IL-1α, IL-1β, IL-6, and IL-17 [23], thereby reducing senescence and extending lifespan. Consequently, the downregulation of inflammatory cytokines affords protection against systemic inflammation, including shielding against neuroinflammation and synaptic loss, through the deactivation of microglia-mediated inflammation and activation of the NOX2–NF-kB pathway. Administration of high doses of taurine in rat models of intracerebral hemorrhage (ICH) has been demonstrated to ameliorate white matter injury and neuronal damage by suppressing inflammatory mediators, glial activation, and neutrophil infiltration while concurrently enhancing CBS expression [72,73]. Huf and co-workers revealed that the effect of taurine supplementation shows promise in alleviating cognitive impairment across diverse conditions and examined taurine’s role in various cognitive impairments and its effects on cognition in aging, Alzheimer’s disease, streptozotocin-induced brain damage, ischemia, mental disorders, genetic diseases, and drug/toxin-induced cognitive deficits [71]. The evidence suggests that taurine may enhance cognitive function through diverse mechanisms, underscoring its potential as a therapeutic agent for cognitive disorders [74]. Taurine also exerts inhibitory effects on microglia-mediated neuroinflammation in paraquat-induced Parkinson’s disease (PD) models. In Alzheimer’s disease (AD), taurine increases the population of reactive astrocytes, thus limiting Aβ-induced inflammation. Furthermore, it exhibits promise for attenuating neurotoxic injury in amyotrophic lateral sclerosis (ALS) cell line models [75].

However, studies on the neuroprotective effects of taurine against ethanol are scarce, and, so far, they have been limited to the analysis of the cerebellum in neonatal mice [52,53]. These studies demonstrated the neuroprotective action of taurine in the cerebellar cells of neonatal mice subjected to acute ethanol intoxication. There was a significant reduction in the number of cells immunopositive for the apoptosis markers Caspase-3 and Tunel in various cerebellar regions after acute taurine administration in ethanol-intoxicated animals. However, they also found that taurine did not protect Purkinje cells in 4-day-old animals. This suggests that the neuroprotective effects of taurine are not linear, varying according to the neuronal type studied and the concentration of taurine in the plasma.

The results showed a trend towards increased cell proliferation and cell survival in the EtOH/TAU group compared to the H_2_O/TAU group. However, overall, the H_2_O/TAU and EtOH/TAU groups did not exhibit statistically significant differences. These groups also did not show significant differences compared to the H_2_O/Sal group. The only statistically significant difference was observed in cell survival in the GCL/SGZ between the two groups. This is likely due to the higher standard deviation commonly observed in in vivo studies.

These results were unexpected but can be explained by the properties of taurine in relation to ethanol exposure. The endogenous taurine system may be an important modulator of ethanol’s effects on the nervous system due to its crucial role in physiological processes such as osmoregulation, neuroprotection, and neuromodulation [52,76]. The results of this manuscript suggest an important interaction between ethanol and taurine that requires further investigation.

The number of pyknotic cells in the H_2_O/TAU group was significantly higher compared to the H_2_O+Sal group. As cell proliferation and cell survival were not affected, these data suggest that taurine may be associated with the removal of non-viable cells or cells that would not become fully functional neurons via apoptosis [77].

The results observed in our experiment showed that after 28 days following the cessation of ethanol consumption, cell proliferation returned to the parameters of the control group animals, who received only water. This likely occurred due to the fact that after ethanol exposure, during the abstinence period, there is a natural brain recovery process, both in humans [78] and animals [27,28]. Although this mechanism is not yet fully understood, it may provide important insights into this endogenous self-repair process. Studies have shown that this neuronal self-repair can be positively influenced by various factors, such as physical exercise [79] and enriched environments [80]. Animals in the EtOH/TAU group did not show statistically significant differences when compared to animals in the H_2_O/Sal and EtOH/Sal groups, suggesting that 28 days after ethanol consumption, taurine had no effect.

Chen et al., and Rivas-Aranciba et al. [74,81] demonstrated that taurine administration promoted cognitive improvements. Rivas-Aranciba et al. showed that, young, adult, or elderly animals exposed to ozone, which induces significant oxidative stress, could be reversed by taurine administration. The same study also showed that control animals, not exposed to ozone but receiving taurine, did not exhibit changes in cognitive tests compared to control animals receiving saline injections. Other studies have shown similar results, where taurine improved hippocampal neurogenesis after lipopolysaccharide injection [82] or in elderly animals [83], but did not induce changes in control groups or healthy young animals, respectively. It is known that the use of isoflurane, an anesthetic that can induce cognitive deficits, especially in elderly patients after surgery, can have detrimental effects. Therefore, Zhang et al. [45] found that pre-treatment with taurine in elderly rats subjected to isoflurane prevented cognitive dysfunction in these animals by inhibiting apoptosis in the hippocampus. Thus, according to these data, it is likely that taurine does not stimulate neurogenesis or promote cognitive improvements in healthy adult brains, but only in situations where there is already a pre-existing deficit.

According to Kim et al. [84] inflammation triggers the halogenation of taurine via the myeloperoxidase (MPO) system in phagocytes, forming taurine chloramine (TauCl). TauCl inhibits the production of inflammatory mediators and boosts antioxidant protein expression in macrophages. This process aids in resolving inflammation by reducing proinflammatory cytokines and reactive oxygen metabolites while enhancing antioxidant defense.

In astrocytes, Seoul and co-workers [85] demonstrated that Tau-Cl elevates nuclear translocation of nuclear factor E2-related factor (Nrf2) expression, regulating Nrf2-controlled antioxidant enzymes such as heme oxygenase 1 (HO-1), NAD(P)H:quinone oxidoreductase 1 (NQO1), glutamate–cysteine ligase catalytic (GCLC), and glutamate–cysteine ligase modifier (GCLM) via the Kelch-like ECH-associated protein 1 (Keap1) pathway. This action rescues cells from oxidative death induced by H_2_O_2_, enhancing HO-1 expression and suppressing ROS production in the brain, thereby promoting neuroprotection. Perhaps the same mechanism of taurine neuroprotection could occur against alcohol hippocampus injury, since the findings support our results, which show significant effects in the brains of rats following taurine treatment. However, new studies need to be conducted to confirm this hypothesis.

**Figure 9:**
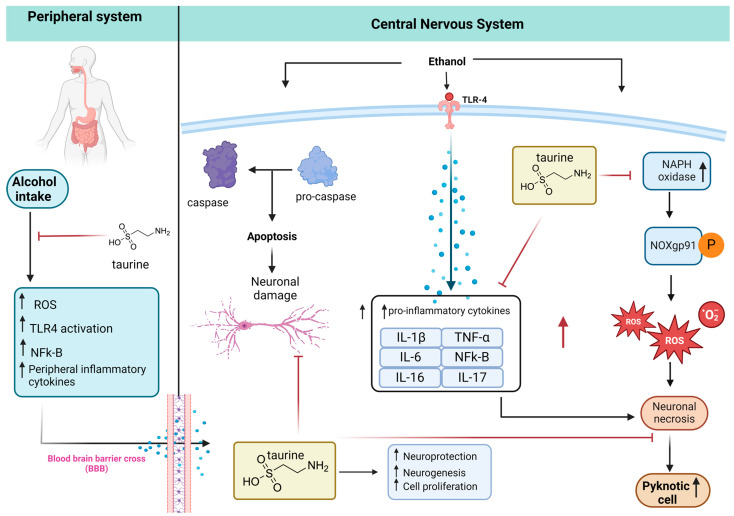
Taurine neuroprotection. The peripheral inflammation caused by alcohol intake increases proinflammatory cytokines that cross the BBB to the CNS. The alcohol in the CNS promotes apoptosis and neuronal damage via the caspase pathway. Alcohol ingestion also activates the expression of Toll-like receptor 4 (TLR-4), increasing the inflammatory cytokines that potentiate the neuronal necrosis caused by ROS produced by NADPH-dependent oxidase (NOX), NOX gp91^phox^ expression, and increasing pyknotic cells. Taurine inhibits peripheral inflammation, neuronal damage, and death (adapted from [11], created with biorender).

Based on the literature review, Figure 9 shows a possible mechanism of neuroprotection of taurine in damaged brains after alcohol consumption. Taurine can inhibit peripheral inflammation by decreasing the blood–brain barrier (BBB) transporter of inflammatory cytokines to the brain, increasing neuroprotection. Kamal et al. [8] review alcohol use disorder, neurodegeneration, and diseases such as Alzheimer and Parkinson. Prolonged and excessive alcohol intake increases ROS, LPO, protein and DNA damage, mitochondrial dysfunction, elevated cytokine production, and ultimately neuronal cell death, with potential links to neurodegenerative diseases.

All research is not entirely conclusive, but it raises questions that open minds to new studies, especially to explain the gaps. This research has a very important result, showing that taurine, a potent antioxidant, has the ability to protect hippocampal neurons and promote neurogenesis during a stressful process like chronic ethanol consumption, but not in normal situations. This suggests that taurine may trigger a process of neuroplasticity activated during the deleterious activity on the hippocampus.

In clinical research and potential human treatments, taurine exerts effects not only in the brain but also in the liver [86], heart [87], and vascular system [88]. These data, despite being in vivo, indicate the potential for human research using taurine as a damage-reversing agent, given its very low associated toxicity, which makes it suitable for clinical research. Similarly, acamprosate, or Campral^®^ (calcium acetyl-homotaurine), a synthetic derivative of taurine, has been used to treat alcohol dependence since 1989; it was first introduced Latin America and Europe [89].

Acamprosate modulates N-methyl-D-aspartate (NMDA) receptor transmission and may indirectly affect γ-aminobutyric acid type A (GABA-A) receptor transmission. It has been shown to decrease brain glutamate levels and increase β-endorphins in both rats and humans. Although acamprosate is recognized as an anti-craving medication, its subjective effects are subtle, primarily alleviating anxiety and insomnia. The effect of acamprosate on NMDA receptors appears to be indirect. Early electrophysiological studies revealed that acamprosate treatment results in increased NMDA receptor-mediated excitation in the nucleus, accumbens, and hippocampus. This suggests that acamprosate may influence NMDA receptor activity through interactions with other neurotransmitter systems rather than directly binding to the receptors [90,91,92], although these effects align with preclinical findings of reduced withdrawal symptoms in animals treated with acamprosate.

However, the combination of alcohol consumption with taurine requires careful attention (e.g., energetic drinks containing taurine in conjunction with alcohol). Considering that both compounds have GABAergic activities, a food/alcohol interaction could occur, potentially causing CNS depression or a drop in glucose level in humans. Given that taurine can either increase or decrease voluntary alcohol intake, depending on the concentrations, timing, and administration pathway, it can induce anxiolytic behavior in rats [93,94] and lead to death in animals after high amounts of alcohol and taurine due to severe hypoglycemia [52].

## 5. Conclusions

In summary, the chronic ethanol consumption model proved effective in promoting a reduction in hippocampal neurogenesis, along with decreases in cell survival and body volume, suggesting significant impairment for animals subjected to this model. Taurine administration emerged as a crucial factor in attenuating and reversing the deleterious effects resulting from ethanol exposure. Analyses of cell proliferation, survival, apoptosis, and neurogenesis in the dentate gyrus revealed that taurine administration provided neuroprotection against ethanol-induced effects. Upon examining the impact of taurine on reversing ethanol’s effects on hippocampal neurogenesis, it was observed that the hippocampus of the animals returned to baseline values of cell proliferation, neurogenesis, and apoptosis after 28 days without ethanol, suggesting a complex mechanism of neuronal self-repair. These findings suggest that taurine can act as a reparative adjunct molecule against the negative effects of alcohol during its consumption but does not induce neurogenesis after ethanol consumption.

## Figures and Tables

**Table 1:** Effect of taurine on hippocampal neurogenesis: Profile of the number of doublecortin (DCX) immunoreactive cells in the dentate gyrus of rats subjected to chronic ethanol consumption (n = 6).

	Groups			
	H_2_O/Sal	EtOH/Sal	H_2_O/TAU	EtOH/TAU
Total	679 ± 56	411 ± 43 ***	546 ± 7 *	580 ± 119 ^###^
Rear	267 ± 55	209 ± 56	321 ± 27	260 ± 59
Front	412 ± 3	202 ± 15 **	225 ± 21	321 ± 90 ^#^

Values found on average ± SE. * *p* < 0.05 vs. H_2_O/Sal, ** *p* < 0.01 vs. H_2_O/Sal, *** *p* < 0.001 vs. H_2_O/Sal. ^#^ *p* < 0.05 vs. EtOH/Sal, ^###^ *p* < 0.001 vs. EtOH/Sal. Statistical analysis: Two-way Anova and Bonferonni. SE = standard error.

## Data Availability

The original contributions presented in the study are included in the article, further inquiries can be directed to the corresponding author.
